# Study - International multicentric minimally invasive liver resection (SIMMILR-4) : a comparison of conventional, digitally enhanced and collaborative robotic with Tele-robotic laparoscopic surgery for colorectal liver metastases

**DOI:** 10.1007/s11701-025-03040-7

**Published:** 2025-12-11

**Authors:** Andrew A. Gumbs, Mohammad Abu-Hilal, Roland Croner, David Fuks, Zacharias Heger Londono, Hadrien Tranchart, Joseph Derienne, Amir Nour Mohammadi, Vincent Grasso, Soufyan el Abdel, Karol Rawicz-Pruzynski, Ibrahim Dagher

**Affiliations:** 1 Hôpital Antoine Béclère, Assistance Publique-Hôpitaux de Paris, 157 Rue de la Porte de Trivaux, Clamart, 92140 France; 2 Department of General-, Visceral-, Vascular- and Transplantation Surgery, University of Magdeburg, Magdeburg, Germany; 3 Department of Surgical Oncology, Medical University of Lublin, Radziwiłłowska 13 St, Lublin, 20-080 Poland; 4 Department of Surgery, School of Medicine, The University of Jordan, Amman, 11942 Jordan; 5 Department of Surgery, University Hospital Southampton NHS Foundation Trust, Southhampton, SO16 6YD UK; 6 Department of Visceral Surgery, CHUV – UNIL, Rue du Bugnon 46, Lausanne, 1005 Switzerland; 7 Department of Electrical and Computer Engineering, University of New Mexico, Albuquerque, NM 87106 USA; 8 Department of Medical Education and Scholarship, Rowan-Virtua School of Osteopathic Medicine, Rowan University, Stratford, NJ 08084 USA; 9 Department of Surgery, Amsterdam UMC, University of Amsterdam, Amsterdam, The Netherlands; 10 Cancer Center Amsterdam, Amsterdam, The Netherlands; 11 Department of General, Visceral, Vascular and Transplant Surgery, University Hospital Magdeburg, Leipziger Str. 44, 39120 Magdeburg, Germany

**Keywords:** Conventional laparoscopy, Digitally enhanced laparoscopy, 3D laparoscopy, Collaborative robotic laparoscopy, Tele-robotic laparoscopic surgery, Remote surgery, Colorectal, Liver metastases, Liver resection, Hepatectomy, Cobotics

## Abstract

This international multicenter study compared five surgical approaches for resection of colorectal liver metastases (CRLM): open, conventional laparoscopy (L), digitally enhanced laparoscopy with 3D imaging (DEL), collaborative robotic laparoscopy with a single robotic arm (CRL) with tele-robotic laparoscopic surgery with 3D imaging and 4 robotic arms (TRL). This exploratory study aimed to assess whether TRL liver resection for colorectal liver metastases (CRLMs) demonstrates potential advantages over conventional laparoscopy (L), digitally enhanced laparoscopy (DEL), or collaborative robotic laparoscopy (CRL). A retrospective analysis of 1,257 patients undergoing CRLM resection across five centers was performed. Surgical techniques were classified as O, L, DEL, CRL, or TRL. Because of limited case numbers in the DEL, CRL, and TRL groups, the primary comparison was between all minimally invasive surgery (MIS) cases combined (L, DEL, CRL, TRL; *n* = 283) and matched open controls (*n* = 283) using 1:1 propensity score matching. Outcomes included estimated blood loss (EBL), operative time, length of stay (LOS), resection margin status (R0), and major complications (Clavien-Dindo grade ≥ 3). MIS demonstrated significantly lower EBL (505 mL vs. 692 mL, *p* < 0.0001) and shorter LOS (8 vs. 14 days, *p* < 0.0001) compared with open surgery. Subgroup analyses of DEL, CRL, and TRL (each < 21 patients post-match) showed comparable perioperative outcomes, suggesting incremental adoption of advanced technologies did not compromise safety or efficiency. MIS offers significant reductions in blood loss and hospital stay compared with open surgery. While TRL provides technical advantages, it did not show clear superiority. Prospective standardized multicenter studies are needed to confirm these exploratory findings.

## 1. Introduction

The SIMMILR (Study - International Multicentric Minimally Invasive Liver Resection**)** project was started to try and better understand the real advantages and potential disadvantages of the complete robotic surgical systems (CRSS) [1]. As we continued to do studies, it became clear to us that many liver surgeons are reporting their results as “laparoscopic” when, in fact, they were actually using aspects of robotics [2]. Because of this observation, we decided to embark on this current study in an effort help surgeons better understand what robotic surgery is according to an engineering perspective.

Mechatronics is a broad interdisciplinary field that encompasses electrical, control and mechanical engineering and computer science. Mechatronics deals with the optimization and design of intelligent products and systems. While all robots are examples of mechatronic systems, not all mechatronic systems are robots. This is because robotic systems also include autonomous actions or decision making. Specifically, they must have, at least, one of the following: (1) sensors that can sense their environments, (2) the ability to process data and make decisions based on the information and/or (3) the capability to act independently. This definition comes from the International Organization for Standardization (ISO 8373:2012 Robots and robotic devices-Vocabulary) and defines a robot as an “actuated mechanism programmable in two or more axes with a degree of autonomy, moving within its environment, to perform intended tasks.”

The exponential advancements in the field of artificial intelligence (AI) have forced surgeons to re-evaluate previous definitions of robotic-assisted surgery (RAS)/robotic surgery [3, 4]. Current CRSSs(e.g. da Vinci, Intuitive Surgical, Sunnyvale, California, USA; Mantra, SSInnovations, New Delhi India; Hugo RAS system, Medtronic Dublin, Ireland) are just remote-controlled systems where the operating surgeon controls the arms and there is essentially only level one surgical autonomy, e.g. telemanipulation only [5]. Notably, the more recent da Vinci robots can be attached to an intelligent operating table that autonomously moves the robotic arms during repositioning of the table (TruSystem 7000dV Surgical Table, TRUMPF Medizin Systeme GmbH & Co, Saalfeld, Germany), and can also use a gastro-intestinal stapler that autonomously adjusts compression speed based on sensor readings on tissue thickness (SmartFire technology, Sure Form staplers, Intuitive Surgical, Sunnyvale, California, USA) [6]. Although, these are early and limited examples of robotics, studies comparing open, laparoscopic and RAS approached, often ignore the subtleties of what robotic surgery is.

When comparing RAS to laparoscopy, the current understanding of RAS is that although it is technically a form of minimally invasive surgery like laparoscopy, it also has 3-Dimensional (3D) imaging, articulating instruments with 7 degrees of freedom, one robotic arm holding the camera and 3 additional robotic arms. The reality is that we do not know which of these additional features provides any benefit. In many ways, we have skipped many steps in the evolution towards robotic surgery. This is perhaps best understood when we look at robots that also have autonomous laparoscope tracking (Senhance, Asensus Surgical, Inc, Durham, North Carolina, USA) and handheld surgical robots [7, 8]. In essence, it could be argued that initial “robotic” surgeons essentially brought a “bazooka to a knife fight,” without properly evaluating all aspects of CRSSs.

Because of this, we used liver resection for CRLM, as a model to try and dissect what actual benefits arise from the utilization of current CRSS. The initial SIMMILR (Study: International Multicentric Minimally Invasive Liver Resection) was for Colorectal Liver Metastases (SIMMILR-CRLM) was a propensity score matched (PSM) study that reported short-term outcomes of patients with CRLM who met the Milan criteria and underwent either open, laparoscopic or robotic liver resection [1]. SIMMILR-2, reported the long-term outcomes from that initial study (now referred to as SIMMILR-1) [1, 9]. A subsequent study analyzed data from 3 international centers doing RAS liver resection for CRLM vs. 1 center that did laparoscopic liver resection with a robotically-controlled laparoscope holder (VidendosKopY, Endocontrol, Grenoble, France) and also using a handheld autonomous gastrointestinal (GIA) handheld stapler device (Signia, Medtronic, Dublin, Ireland) [7, 8]. This study, which should probably be now known as SIMMILR-3 inspired this current study, SIMMILR-4.

RAS is best used to define devices that enable level 1 surgical autonomy (e.g., telemanipulation, local or remote). Telemanipulator surgery is when the operating surgeon operates away from the OR table via a remote-controlled system. Collaborative robotic or cobotic surgery refers to surgical procedures performed through active collaboration between a surgeon and a robotic system that could have partial autonomy and thus corresponds to levels 2 to 4 on the surgical autonomy scale. In cobotic surgery, decision-making and task execution are shared, with the robot contributing perceptual, cognitive, or motor functions to assist, guide, or perform parts of the procedure, while the surgeon retains supervisory control and clinical judgment. Robots are defined as devices that can replicate or replace human actions. As a result, robotic surgery implies any autonomy from the surgical robot, therefore, surgical autonomy levels 2–5 encompass the concept of robotic surgery.

Currently, we have identified 5 ways of doing liver resection : open, conventional laparoscopy (L), digitally enhanced laparoscopy with 3D imaging (DEL), collaborative robotic laparoscopy with a single robotic arm (CRL) with tele-robotic laparoscopic surgery with 3D imaging and 4 robotic arms (TRL). The term TRL was chosen to highlight the fact that RAS is still a form of laparoscopy and that it has an element of distance from the patient, it is by definition a form of remote surgery.

**Fig. 1:**
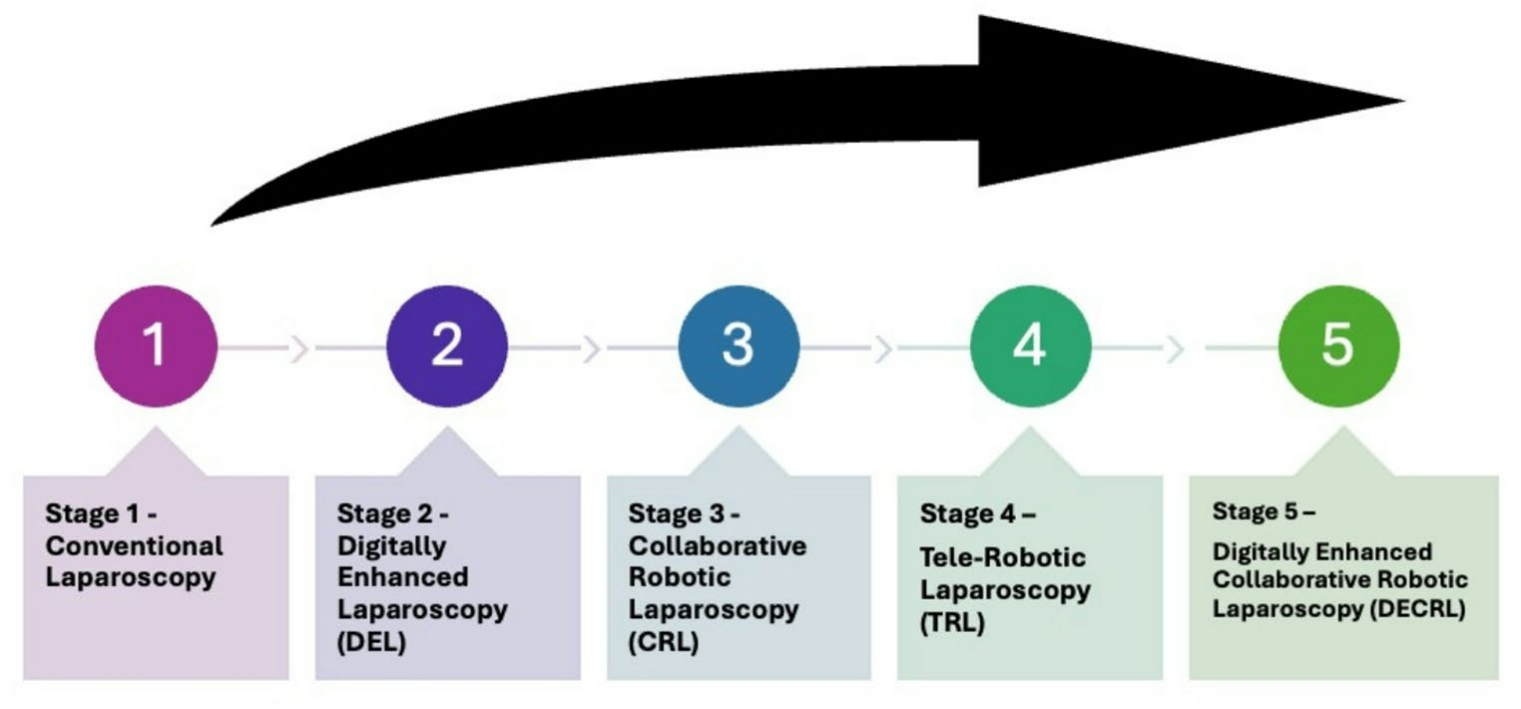
Evolution of minimally invasive surgery from conventional laparoscopy to digitally enhanced collaborative robotic laparoscopy (DECRL). The five stages represent progressive integration of digital imaging, robotic assistance, and remote and local manipulation

To better characterize and distinguish the full spectrum of minimally invasive liver surgery, we organized the approaches into four progressive stages of MIS and theorized about a possible future fifth stage, which perhaps should have been evaluated before TRL (Fig. 1). Stage 1, Conventional Laparoscopy (L), represents the foundational form of MIS, characterized by the use of rigid instruments and a two-dimensional optical system. All camera and instrument movements are manually controlled by the surgeon and assistant, with limited ergonomic and visual support. Stage 2, Digitally Enhanced Laparoscopy (DEL), is a modern evolution of conventional laparoscopy that integrates advanced digital visualization and imaging technologies without robotic actuation. DEL typically includes 3D or 4 K imaging, fluorescence (ICG) guidance, augmented-reality overlays, and artificial intelligence-assisted navigation, while the surgeon retains direct manual control of all instruments.

Stage 3, Collaborative Robotic Laparoscopy (CRL), represents a transitional phase between conventional and fully robotic surgery. In this stage, the surgeon performs conventional laparoscopic maneuvers but is assisted by robotic or motorized systems that aid in camera positioning, instrument stabilization, or ergonomic optimization. These systems function as collaborative robots (cobots), working alongside the surgeon at the patient’s side without full telemanipulation. Stage 4, Tele-Robotic Surgery (4RA), is currently the most advanced stage, in which the surgeon operates from a remote console using telemanipulated robotic arms that replicate hand and wrist movements with precision. Platforms such as the da Vinci, Hugo RAS, and Versius systems exemplify this stage, offering enhanced dexterity, 3D visualization, and tremor filtration while physically separating the surgeon from the operative field. A future stage, Stage 5, might involve Digitally Enhanced Collaborative Robotic Laparoscopy (DECRL) where a surgeon could have all the potential advantages of 4 robotic arms, articulating instruments and 3D imaging, but not be remote and would remain local and in contact with the actual patient.

By comparing the short-term outcomes of these different approaches used in complex liver surgery, with an emphasis on the 4 Stages of technological evolution in minimally invasive liver surgery that currently exist, we hope to better understand the direction that future development in cobotic (local) and tele-robotic (remote) surgery should take. It is theorized that smaller collaborative laparoscopic robotics, may reduce costs and liberate more research dollars for further advancements in AI and autonomous actions in surgery.

## 2. Methods

The primary objective of this study was to compare outcomes among DEL, CRL, and TRL to determine whether tele-robotic laparoscopy provides advantages beyond standard and cobot-enhanced laparoscopic techniques. Comparisons between MIS and Open approaches are presented only as contextual information to demonstrate baseline consistency with prior literature and are not intended to represent a primary endpoint of the study.

### 2.1. Patient selection & indication for surgery

We collected perioperative data as well as follow-up data of all eligible patients who underwent Open or MIS for resection of colorectal metastasis between June 2004 and November 2024. All hepatectomies for colorectal liver metastases were done by surgeons with experience in both open and minimally invasive liver resection at 5 different international centers (2 in France, 1 in Switzerland, 1 in Germany, and 1 in Jordan). Minimum case requirements for participating HPB surgeons in this study was the completion of at least, 50 laparoscopic and/or robotic hepatectomies and, at least, 50 hepatectomies for CRLM.

Surgery was indicated for resectable liver metastases, either synchronous or metachronous, in colorectal cancer patients. Histological confirmation was obtained before surgery when needed. Otherwise, clear imaging evidence of liver metastasis was sufficient. However, to ensure patient-centered care, treatment options—perioperative, surgical, or alternative—were reviewed by a multidisciplinary tumor board (MDT) beforehand. Absolute contraindications to minimally invasive liver surgery included closed-angle glaucoma and intracranial hypertension. Severe lung disease was considered a relative contraindication.

Patients were divided into 5 approaches: open, conventional laparoscopy (L), digitally enhanced laparoscopy with 3D imaging (DEL), collaborative robotic laparoscopy with a single robotic arm (CRL) with tele-robotic laparoscopic surgery with 3D imaging and 4 robotic arms (TRL). In this study, the term “digitally enhanced laparoscopy” refers exclusively to 3D laparoscopy and does not include additional imaging modalities such as ICG fluorescence, AI overlays, or 4 K visualization. In this study, TRL refers to tele-robotic laparoscopy, defined as telemanipulated laparoscopic surgery performed with a complete four-arm robotic platform. This terminology is intentional. From an engineering standpoint, modern “robotic” or “robotic-assisted” surgery remains a form of laparoscopy, because the surgeon operates laparoscopic instruments remotely through a telemanipulation interface. Much of the confusion in the current literature arises from the use of the term “robotic” without acknowledging that it is still laparoscopic surgery. Our classification highlights this point by explicitly framing telemanipulated systems as a subtype of minimally invasive laparoscopy rather than as a separate category.

Patients were taken from 5 centers, 4 in Europe and 1 in the United States. All centers did open and L resection, 3 centers did TRL resections, 1 center did DEL resections and the 1 center that did CRL also utilized handheld robotic GIA staplers. Because some surgeons using the TRL system did not have access to the Sure Form staplers (SmartFire technology, Sure Form staplers, Intuitive Surgical, Sunnyvale, California, USA) we only emphasized the solitary robotic arm (CRL) for the name of this group. A total of 10 head-to-head comparisons were analyzed. (1) O vs. L, (2) O vs. DEL, (3) O vs. CRL, (4) O vs. TRL, (5) L vs. DEL, (6) L vs. CRL, (7) L vs. TRL (8) DEL vs. CRL, (9) DEL vs. TRL and (10) CRL vs. TRL. Unlike SIMMILR-1,2 and 3, the low patient numbers of patients undergoingDEL and CRL, prevented us from limiting resections to patients within the Milan Criteria for SIMMILR-4.

Due to the low numbers in the DEL, CRL and TRL groups, comparisons of groups were ultimately divided into 3 categories : (1)open vs. minimally invasive surgery (MIS), including all patients in these groups L, DEL, CRL and TRL); (2) a comparison of Digitally Enhanced Laparoscopy consisting of laparoscopy (L) vs. DEL and DEL vs. TRL because both modalities use 3D imaging and lastly comparison between procedures done with 1 robotic arm (CRL) vs. 4 robotic arms (TRL).

Because the dataset spans 2004–2024 across five international centers, we anticipated heterogeneity in imaging systems, anesthesia care, perioperative workflows, and evolving surgical technologies. To minimize this variability, standardized variable definitions were applied across centers, and propensity score matching included multiple confounders related to patient and disease complexity. However, residual heterogeneity in perioperative management and technological evolution could not be fully eliminated and is acknowledged as a study limitation.

### 2.2. Study endpoints

A retrospective analysis was conducted on patients who received liver resection for colorectal metastases. Individuals who had prior Associating Liver Partition and Portal Vein Ligation for Staged Hepatectomy (ALLPS), ablation therapy, or repeat resections were excluded. Cases initiated with a minimally invasive technique were assessed on an intention-to-treat basis. Study data can be accessed from the corresponding author upon justified request.

Demographics and confounding variables used for the propensity score matching (PSM) included : age, sex, American Society of Anesthesia (ASA) class, BMI (Body Mass Index) calculated as kg/m², history of previous surgery, neoadjuvant therapy (e.g. chemotherapy, chemoradiotherapy and/or immunotherapy, largest metastasis size in mm, number of metastases, location in deep segments and type of liver resection (Major or Minor). Deep segments were defined as lesions in segments 1, 4 A, 7 and 8. The main outcome measured was short-term mortality (death within 30 or 90 days post-surgery). Secondary measures included operative factors (blood loss, OR duration), hospitalization period, R0 resection status, and major complications. The Dindo-Clavien system classified postoperative issues, with severe cases defined as grade ≥ 3. PSM ensured better technique comparison.

Written informed consent was acquired from all participants, specifying that anonymized data might be used in later research. The STROBE checklist guided manuscript preparation for observational study reporting. Surgical techniques and definition of extent of liver resection have been previously described in SIMMILR 1–3.

### 2.3. Data analysis

Statistical evaluation was conducted using Social Science Statistics software (www.socscistatistics.com, accessed 22 December 2021) and SPSS (v26; IBM, Armonk, NY). Categorical variables (nominal/ordinal) were shown as counts (n) and/or percentages (%). Group comparisons used Pearson’s χ² test or Fisher’s exact test (if any cell had < 5 observations). Continuous measures were reported as mean ± SD.

Continuous variables were expressed as means ± standard deviations (SD) and compared using the Student’s t-test or Mann-Whitney U test, as appropriate. Categorical variables were presented as frequencies and percentages and analyzed using the chi-square test or Fisher’s exact test. Effect sizes were calculated and reported as mean differences (Δ) for continuous variables and odds ratios (OR) for categorical variables, each with corresponding 95% confidence intervals (CI) to enhance clinical interpretability. All statistical tests were two-tailed, and a p-value < 0.05 was considered statistically significant. Data analysis was performed using SPSS version 28.0 (IBM Corp., Armonk, NY, USA) or R version 4.3.1 (R Foundation for Statistical Computing, Vienna, Austria).

### 2.4 Propensity score matching (PSM)

**Fig. 2:**
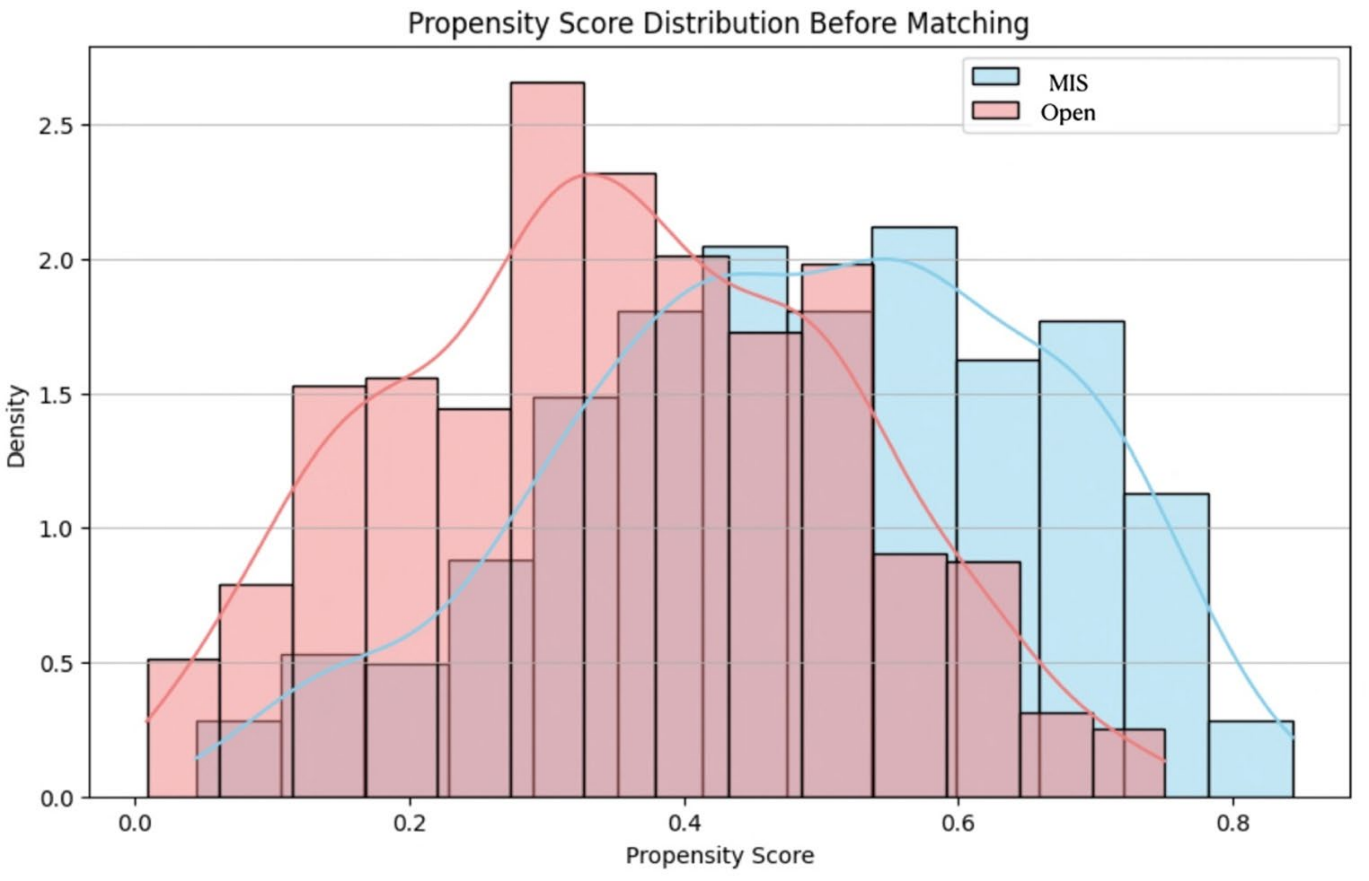
Propensity Score Distribution Before Matching. This figure displays the distribution of estimated propensity scores for patients undergoing the Open liver resection (Open group) versus Minimally Invasive Surgery (MIS group) consisting of Conventional Laparoscopy (L), Digitally Enhanced Laparoscopy (DEL), Collaborative Robotic Laparoscopy (CRL) and Tele-Robotic Laparoscopy (TRL) prior to propensity score matching. Propensity scores were calculated using a logistic regression model with age, gender, ASA score, BMI, tumor type, margin status, tumor size, number of tumors, and location in deep segments as covariates. The disparity in the distributions indicates significant baseline differences in observed confounders between the groups before matching

PSM was performed to reduce confounding by indication in this retrospective observational study (Fig. 2). PSM is a robust statistical method commonly used in non-randomized settings to emulate some of the balance achieved in randomized controlled trials. In this analysis, the propensity score was estimated using a logistic regression model, where treatment assignment served as the dependent variable and relevant covariates—including demographic, clinical, and procedural characteristics—were included as independent variables. The goal was to derive a summary score reflecting each patient’s probability of receiving the treatment, conditional on the observed covariates.

**Fig. 3:**
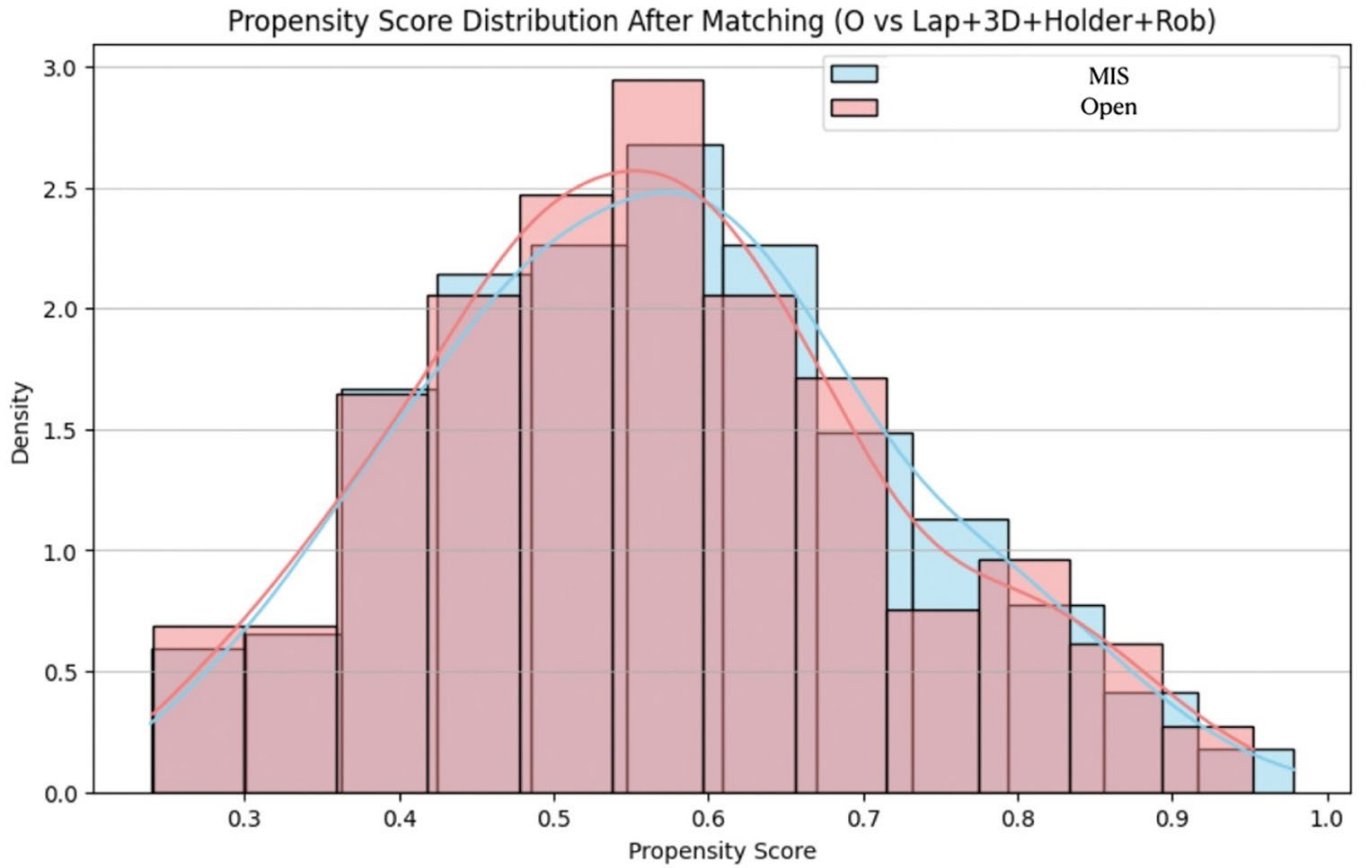
Propensity Score Distribution After Matching. This figure illustrates the distribution of estimated propensity scores in the propensity score-matched group from the Open (Open group) versus Minimally Invasive Surgery (MIS group) consisting of Conventional Laparoscopy (L), Digitally Enhanced Laparoscopy (DEL), Collaborative Robotic Laparoscopy (CRL) and Tele-Robotic Laparoscopy (TRL). Propensity score matching was performed using a 1:1 nearest-neighbor algorithm without replacement and a caliper of 0.2. The substantial overlap and similar distributions of propensity scores between the matched groups demonstrate that the matching procedure effectively balanced the distribution of observed confounders, allowing for more robust comparisons of outcomes

After estimating the propensity scores, a nearest-neighbor matching algorithm without replacement was employed to form matched pairs (Fig. 3). A caliper width of 0.2 times the standard deviation of the logit of the propensity score was used to ensure tight matching, thereby limiting residual imbalance. Patients in the treatment group were matched to those in the control group with similar propensity scores, and unmatched subjects were excluded from the final analysis. Standardized mean differences (SMDs) were calculated before and after matching to assess covariate balance; an SMD less than 0.1 was considered indicative of adequate balance.

Matching diagnostics and visualization techniques, including density plots and Love plots, were used to further validate the adequacy of the matching procedure. Post-matching comparisons of outcomes between groups were conducted using appropriate statistical tests, accounting for the matched design. All analyses were performed using Python with the pymatch and statsmodels libraries, and the entire procedure adhered to best practices in observational comparative effectiveness research.

This methodological approach was selected to minimize bias from confounding variables and strengthen causal inference regarding the association between the surgical intervention and outcomes. By ensuring that matched groups were well balanced across baseline characteristics, the risk of spurious associations due to selection bias was substantially reduced.

## 3. Results

Tables 1, 2 and 3 show the demographics, confounding and short-term outcome variables for liver resection for colorectal liver metastases after propensity score matching, SIMMILR-4 (Study - International Multicentric Minimally Invasive Liver Resection). These tables showed the demographics and confounding variables. Tables 4, 5 and 6 showed the short-term outcome variables. All centers in this study that did TRL used the da Vinci robot (da Vinci, Intuitive Surgical, Sunnyvale, CA, USA).

**Fig. 4:**
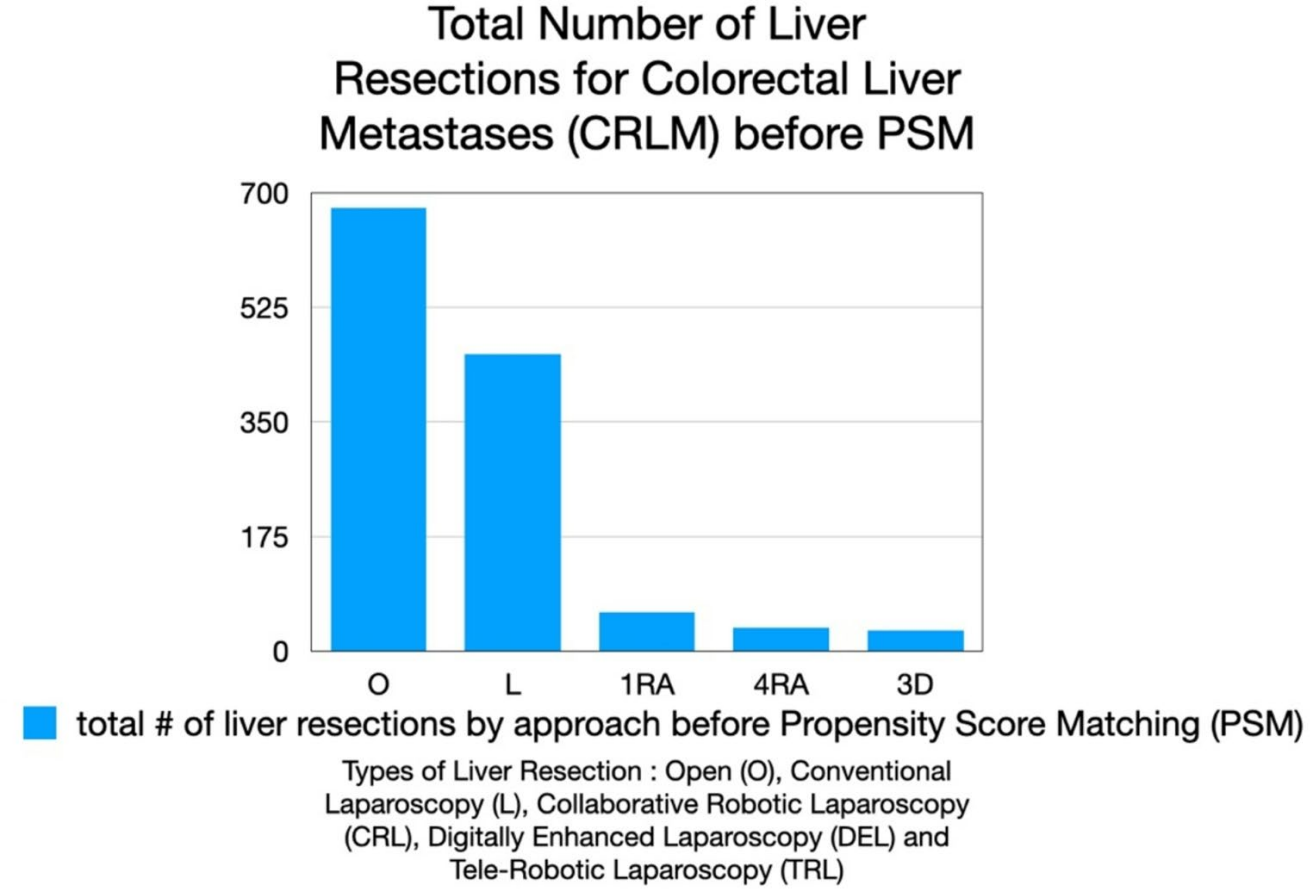
Evolution of minimally invasive surgery from current Conventional Laparoscopy (L), Digitally Enhanced Laparoscopy (DEL), Collaborative Robotic Laparoscopy (CRL) and Tele-Robotic Laparoscopy (TRL) to a future of digitally enhanced collaborative robotic laparoscopy (DECRL). The five stages represent progressive integration of digital imaging, robotic assistance, and remote and local manipulation

A total of 1,257 patients undergoing liver resection for colorectal liver metastases were identified from the 5 centers : 677 underwent open resection, 453 conventional laparoscopic (L) resection, 60 laparoscopic resection with a single robotic arm holding the laparoscope and a handheld robotic GIA stapler device (CRL), 36 RAS resection with the CRSS a 4 arm tele-robotic laparoscopic system (TRL) and 31 via laparoscopy with 3D imaging (DEL) (Fig. 4). Tables 1, 2 and 3 found no statistically significant differences after PSM in the preoperative demographics and confounding variables, however, as seen below, some variables approached statistical significance.

In Table 1, resected lesions tended to be in the deep segments more often in the Open group when compared to the MIS group, 71.2% vs. 63.8% (*p* = 0.07), respectively. Additionally, the MIS group tended to undergo more major resections, 48% vs. 42% (*p* = 0.06), respectively. When conventional laparoscopic (L) resection was compared to laparoscopy with 3D imaging (DEL) (Table 2), the DEL group tended to have a higher percentage of patients who had undergone previous abdominal surgery, 95.2% vs. 76.2% (*p* = 0.08), respectively. No tendencies were noted when DEL was compared to the TRL group. When 1 Arm Robotic-Assisted (CRL) liver resection was compared with TRL, patients in the TRL group tended to have higher ASA classification (*p* = 0.06), notably, there were only 9 patients in each of these groups (Table 3).

**Table 1:** Demographics and Confounding Variables after propensity score matching, SIMMILR-4 (Study - International Multicentric Minimally Invasive Liver Resection): Open vs. Minimally Invasive Surgery (MIS) [Conventional Laparoscopy (L)(n=222), 3-D Laparoscopy (3D)(n=15), 1 Arm Robotic-Assisted (1RA) (n=22) and 4 Arm Robotic-Assisted (4RA)(n=24)]

Variable	Open (O) (n=283)	MIS (n=283)	Effect Size (95% CI)	p-value
Age, mean ± SD	63 ± 13.4	62 ± 13.0	Δ = 1.0 yrs (–1.5 to 3.4)	0.9
Male : Female	170 : 112	164 : 119	OR = 1.12 (0.80–1.57)	0.6
ASA III–IV (%)	89 (31.4%)	70 (24.7%)	OR = 1.39 (0.94–2.07)	0.09
BMI (kg/m²), mean ± SD	27.0 ± 4.9	26.0 ± 4.1	Δ = 1.0 (–0.6 to 2.6)	0.2
Previous Surgery (%)	209 (74.0%)	205 (72.6%)	OR = 1.07 (0.72–1.58)	0.8
Neoadjuvant Therapy (%)	147 (51.9%)	175 (59.3%)	OR = 0.74 (0.52–1.06)	0.1
Metastasis Size (mm), mean ± SD	37.2 ± 31.3	29.9 ± 20.6	Δ = 7.3 mm (–1.8 to 16.4)	0.9
Deep Segment Location (%)	183 (71.2%)	164 (63.8%)	OR = 1.40 (0.97–2.03)	0.07
Major Resection (%)	42 (14.8%)	48 (17.0%)	OR = 0.84 (0.54–1.31)	0.06

M=male; F=Female; ASA=American Society of Anesthesia; BMI=Body Mass Index

**Table 2:** Demographics and Confounding Variables after propensity score matching, SIMMILR-4 (Study - International Multicentric Minimally Invasive Liver Resection): Standard laparoscopy (L) vs. Digitally Enhanced Laparoscopy (DEL) [3-D Laparoscopy (3D) and 4 Arm Robotic-Assisted with 3-D imaging(4 RA)]

Variable	Laparoscopy (L) (n=21)	3-D Laparoscopy (3D) (n=21)	Effect Size (95% CI) L vs 3D	p-value (L vs 3D)	3-D Laparoscopy (3D) (n=13)	4 Arm Robotic-Assisted (4 RA) (n=13)	Effect Size (95% CI) 3D vs 4RA	p-value (3D vs 4RA)
Age, mean (range)	70.6 ±8.9	69.2 ±9.0	Δ = 1.4 yrs (–2.1 to 4.9)	0.4	68.8 ±8.5	66.2 ±14.7	Δ = 2.6 yrs (–3.4 to 8.6)	1
Male : Female	10 : 11	10 : 11	OR = 1.00 (0.33–3.01)	1	7 : 6	10 : 3	OR = 0.27 (0.05–1.53)	0.2
ASA III–IV (%)	6 (28.6%)	6 (28.6%)	OR = 1.00 (0.28–3.53)	1	6 (46.2%)	6 (46.2%)	OR = 1.00 (0.23–4.39)	0.8
BMI (kg/m²)	27.1 ±5.7	26.1 ±5.9	Δ = 1.0 (–2.5 to 4.5)	0.3	26.6 ±7.1	21.6 ±5.8	Δ = 5.0 (–1.8 to 11.8)	0.7
Previous Surgery (%)	16 (76.2%)	20 (95.2%)	OR = 0.17 (0.02–1.52)	0.08	12 (92.3%)	10 (77.0%)	OR = 3.00 (0.46–19.6)	0.3
Neoadjuvant Therapy (%)	17 (81.0%)	13 (61.9%)	OR = 2.67 (0.68–10.5)	0.2	8 (61.5%)	5 (38.5%)	OR = 2.56 (0.50–13.2)	0.2
Metastasis Size (mm)	30.4 ±16.7	31.5±22.0	Δ = –1.1 mm (–6.0 to 3.8)	0.6	38.2 ±20.4	34.7 ±8.2	Δ = 3.5 mm (–6.8 to 13.8)	0.7
Number of Tumors	1.34 ±0.6	1.67 ±0.2	Δ = –0.33 (–0.78 to 0.12)	0.08	2 ±0.3	1.8 ±0.6	Δ = 0.2 (–0.4 to 0.8)	0.2
Deep Segment Location (%)	11 (52.4%)	14 (66.7%)	OR = 0.54 (0.14–2.06)	0.3	9 (69.2%)	7 (53.8%)	OR = 1.95 (0.38–10.1)	0.4
Major Resection (%)	4 (19.0%)	6 (28.6%)	OR = 0.59 (0.15–2.34)	0.5	3 (23.1%)	2 (15.4%)	OR = 1.63 (0.23–11.7)	0.6

M=male; F=Female; ASA=American Society of Anesthesia; BMI=Body Mass Index

**Table 3:** Demographics and Confounding Variables after propensity score matching, SIMMILR-4 (Study - International Multicentric Minimally Invasive Liver Resection): Cobotic Laparoscopy (CL) [1 Arm Robotic-Assisted (1 RA)] vs. Robotic surgery [4 Arm Robotic-Assisted (4 RA)]

Variable	1-Arm Robotic-Assisted (1RA) (n=9)	4-Arm Robotic-Assisted (4RA) (n=9)	Effect Size (95% CI)	p-value
Age, mean (range)	60.2 ±15.9	60.1 ±17.0	Δ = 0.1 yrs (–7.5 to 7.7)	0.9
Male : Female	3 : 6	5 : 4	OR = 0.40 (0.07–2.44)	0.3
ASA III–IV (%)	2 (22.2%)	6 (66.7%)	OR = 0.15 (0.02–1.17)	0.06
BMI (kg/m²), mean (range)	31.9 ±3.3	31.1 ±3.1	Δ = 0.8 (–3.0 to 4.6)	0.7
Previous Surgery (%)	6 (66.7%)	6 (66.7%)	OR = 1.00 (0.17–5.78)	1.0
Neoadjuvant Therapy (%)	4 (44.4%)	3 (33.3%)	OR = 1.60 (0.27–9.53)	0.6
Metastasis Size (mm), mean (range)	45.0 ±31.5	30.8 ±10.4	Δ = 14.2 mm (–10.5 to 38.9)	0.67
Number of Tumors, mean (range)	1.2 ±0.3	1.8 ±0.6	Δ = –0.6 (–1.3 to 0.1)	0.09
Deep Segment Location (%)	6 (66.7%)	5 (55.6%)	OR = 1.60 (0.27–9.53)	0.6
Major Resection (%)	3 (33.3%)	2 (22.2%)	OR = 1.71 (0.23–12.5)	0.6

M=male; F=Female; ASA=American Society of Anesthesia; BMI=Body Mass Index

When the short-term outcome variables were studied, multiple significant and highly significant findings were identified, however, due to the low numbers of patients in the DEL, CRL and TRL groups, findings in Tables 5 and 6 are only considered exploratory (Tables 4, 5 and 6). EBL, LOS, Clavien-Dindo morbidity ≥ 3, 90 day mortality and R1 resection rates were all improved in the MIS group when compared to the Open group (Table 4). Ninety-day mortality was lower in the MIS group than in the Open group, and this difference was statistically significant (*p* = 0.006). Similarly, R1 resection rates were lower after MIS compared with Open resection (*p* = 0.002). The findings for the EBL and LOS were extremely statistically significant (*p* < 0.0001) while notably, however, the operative time was lower in the O group and this finding was statistically significant, 270 vs. 257 min, respectively (*p* = 0.03).

**Table 4:** Short term Outcome Variables after propensity score matching, SIMMILR-4 (Study - International Multicentric Minimally Invasive Liver Resection): Open vs. Minimally Invasive Surgery (MIS) [Laparoscopy (L)(n=222), 3-D Laparoscopy (3D)(n=15), 1 Arm Robotic-Assisted (1RA) (n=22) and 4 Arm Robotic-Assisted (4RA)(n=24)]

Variable	Open (O) (n=283)	Minimally Invasive Surgery (MIS) (n=283)	Effect Size (95% CI)	p-value
Estimated Blood Loss (mL), mean (range)	692.2 ±558.0	504.8 ±566.1	Δ = 187.4 mL (110.2–264.6)	<0.0001
Operative Time (min), mean (range)	256.5 ±93.4	269.5 ±99.2	Δ = –13.0 min (–25.0 to –1.0)	0.03
Length of Stay (days), mean (range)	13.6 ±13.9	8.4 ±15.7	Δ = 5.2 days (3.7–6.7)	<0.0001
Conversion Rate (%)	NA	23 (8.4%)	–	NA
Clavien-Dindo ≥ Grade III morbidity (%)	39 (13.8%)	25 (8.9%)	OR = 1.63 (0.93–2.87)	0.11
30-day mortality (%)	14 (5.8%)	8 (2.6%)	OR = 2.33 (0.96–5.63)	0.10
90-day mortality (%)	15 (12.8%)	8 (3.5%)	OR = 3.96 (1.46–10.7)	0.006
R1 Resection (%)	60 (24.4%)	36 (13.4%)	OR = 2.09 (1.31–3.34)	0.002

M=male; F=Female; ASA=American Society of Anesthesia; BMI=Body Mass Index

**Table 5:** Short term Outcome Variables after propensity score matching, SIMMILR-4 (Study - International Multicentric Minimally Invasive Liver Resection): Standard laparoscopy (L) vs. Digitally Enhanced Laparoscopy (DEL) [3-D Laparoscopy (3D) and 4 Arm Robotic-Assisted with 3-D imaging (4 RA)]

Variable	Laparoscopy (L) (n=21)	3-D Laparoscopy (3D) (n=21)	Effect Size (95% CI)	p-value	3-D Laparoscopy (3D) (n=11)	4-Arm Robotic-Assisted (4RA) (n=11)	Effect Size (95% CI).1	p-value
Estimated Blood Loss (mL), mean (range)	424.4 ±471.6	218.8 ±427.9	Δ = 205.6 mL (30.2–381.0)	0.02	636.4 ±658.9	300 ±296.4	Δ = 336.4 mL (–220.1 to 892.9)	1.0
Operative Time (min), mean (range)	198.3 ±115.0	206.2 ±64.7	Δ = –7.9 min (–37.2 to 21.4)	0.7	211.8 ±55.9	356.6 ±131.2	Δ = –144.8 min (–229.7 to –59.9)	0.002
Length of Stay (days), mean (range)	5.1 ±2.6	6.6 ±3.2	Δ = –1.5 days (–3.3 to 0.3)	0.09	10.8 ±5.8	5.7 ±2.9	Δ = 5.1 days (1.5–8.7)	0.008
Conversion Rate (%)	3 (14.3%)	2 (9.5%)	OR = 1.58 (0.24–10.4)	0.6	1 (9.1%)	1 (9.1%)	OR = 1.00 (0.06–16.6)	1.0
Clavien-Dindo ≥ Grade III morbidity (%)	2 (9.5%)	6 (28.6%)	OR = 0.27 (0.05–1.51)	0.1	3 (27.3%)	1 (9.1%)	OR = 3.67 (0.33–40.8)	0.3
30-day mortality (%)	0 (0%)	3 (23.8%)	OR = 0.11 (0.01–2.38)	0.2	2 (18.2%)	0 (0%)	OR = 5.11 (0.23–111.6)	0.5
90-day mortality (%)	0 (0%)	3 (23.8%)	OR = 0.11 (0.01–2.38)	0.2	2 (18.2%)	0 (0%)	OR = 5.11 (0.23–111.6)	0.5
R1 Resection (%)	2 (10.0%)	0 (0%)	OR = 5.22 (0.24–113.2)	0.5	0 (0%)	0 (0%)	–	1.0

EBL = Estimated Blood Loss, OR = Operating Room, LOS= Length of Stay

Table 6 attempted to show what advantages CRL resections had when compared to TRL. Notably, no short-term outcome variable showed any difference that was statistically significant when liver resections for CRLM were done via CRL or TRL, although each group included only nine patients.

**Table 6:** Short term Outcome Variables after propensity score matching, SIMMILR-4 (Study - International Multicentric Minimally Invasive Liver Resection): Cobotic Laparoscopy (CL) [1 Arm Robotic-Assisted (1 RA)] vs. Robotic-Assisted Surgery [4 Arm Robotic-Assisted (4 RA)]

Variable	1-Arm Robotic-Assisted (1RA) (n=9)	4-Arm Robotic-Assisted (4RA) (n=9)	Effect Size (95% CI)	p-value
Estimated Blood Loss (mL), mean (range)	371.1 ±466.4	211.1 ±292.5	Δ = 160.0 mL (–170.0 to 490.0)	1.0
Operative Time (min), mean (range)	217.9 ±147.9	235.7 ±83.3	Δ = –17.8 min (–61.8 to 26.2)	0.3
Length of Stay (days), mean (range)	3.9 ±2.26	4.2 ±2.5	Δ = –0.3 days (–1.5 to 0.9)	0.8
Conversion Rate (%)	0 (0%)	0 (0%)	–	1.0
Clavien-Dindo ≥ Grade III morbidity (%)	0 (0%)	1 (11.1%)	OR = 0.31 (0.01–7.96)	1.0
30-day mortality (%)	0 (0%)	0 (0%)	–	1.0
90-day mortality (%)	0 (0%)	0 (0%)	–	1.0
R1 Resection (%)	0 (0%)	2 (22.2%)	OR = 0.16 (0.01–3.94)	0.5

EBL = Estimated Blood Loss, OR = Operating Room, LOS= Length of Stay

When short-term outcome variables were evaluated, several significant and highly significant differences were observed. However, because the DEL, CRL, and TRL subgroups included relatively small numbers of patients, the findings in Tables 5 and 6 should be interpreted as exploratory. In the overall comparison of MIS versus Open surgery (Table 4), EBL, LOS, Clavien-Dindo morbidity grade 3 or higher, 90-day mortality, and R1 resection rates were all improved in the MIS cohort. Ninety-day mortality and R1 resection rates reached statistical significance (*p* = 0.006 and *p* = 0.002, respectively), while reductions in EBL and LOS were highly significant (*p* < 0.0001). Operative time was shorter in the Open group (270 vs. 257 min), and this difference was statistically significant (*p* = 0.03).

## 4. Discussion

This multi-centric retrospective study, conducted across 5 international centers, analyzed short-term outcomes of various types of minimally invasive liver resection to determine whether, and in what ways, the use of the CRSS offers advantages during removal of CRLMs. To maintain alignment between the study aim and the key findings, the interpretation of results focuses on differences among DEL, CRL, and TRL. The MIS versus Open comparisons are presented as secondary, contextual observations, as the advantages of MIS over Open surgery have already been well established in prior studies. When comparing open and MIS liver resection, 283 patients were included in each group. The advantages of MIS are evident, with improvements across nearly all short-term outcome variables. The only exception was operative time, which was statistically shorter in the open group; however, the absolute difference was small at 13 min (*p* < 0.03) and is unlikely to be clinically meaningful (Table 4). These observations have been noted in other studies, however, a large randomized controlled trial noted no differences in operative times [10, 11]. Furthermore, the potential advantages of MIS liver resection have already been well described, specifically tendency for decrease EBL, LOS but tempered by increased operative times and costs [12–17].

Table 5 attempted to ascertain what DEL brings to the table. To do this, conventional laparoscopy is compared to laparoscopy with 3D imaging (DEL), and DEL is then compared to TRL, which also has 3D imaging. Compared with conventional laparoscopy, DEL showed lower blood loss, but slightly longer hospital stays. When DEL was compared to liver resections with four robotic arms (TRL), operating time was shorter with DEL resections, but hospital stay was longer.

Table 6 compared liver resections using CRL co to liver resection with TRL. No meaningful differences in short-term outcomes were found when comparing liver resections via CRL with TRL resection. Although R1 resection rates seemed to be higher in the TRL group when compared to the CRL group, this difference was not statistically significant. Other studies have shown excellent R0 resection rates with the CRSS [13, 18, 19]. In fact, the University of Magdeburg did a meta-analysis where they found that enhanced R0 rate is an advantage of the robot when compared to conventional laparoscopy, nonetheless, enhancements via cobotics may eliminate this perceived advantage [19].

## 3D imaging

The first and most pressing issue that should probably have received more attention, was how 3D laparoscopy improved short and long-term outcomes after liver resection. Granted, the reluctance to adopt this technology may have been exacerbated by the fact that earlier versions of 3D laparoscopy weren’t as sophisticated as more modern versions. Nevertheless, two-dimensional (2D) laparoscopy presents several challenges, including a lack of depth perception, increased difficulty in managing vascular structures, and a longer learning curve for surgeons [20]. In contrast, 3D laparoscopy has been developed and utilized to overcome these limitations by improving visual clarity and most importantly spatial orientation during surgery. A review of four studies involving 361 patients (159 in the 3D group and 202 in the 2D group) highlighted potential benefits of 3D laparoscopy, particularly during liver resections [21]. Although EBL, reoperation rates, and in-hospital mortality showed no significant differences between the two groups, overall morbidity was found to be lower when 3D laparoscopy was implemented. It is important to note that none of the studies provided details on the learning curve, which may have influenced outcomes.

Some clinical trials suggest that 3D laparoscopy offers shorter operative times, reduced blood loss, and improved precision, especially in complex liver procedures [22, 23]. Surgeons report enhanced depth perception and potentially faster skill acquisition when using 3D systems [24]. These advantages may lead to safer and more efficient operations. However, certain limitations exist, including the higher cost of 3D equipment, the need for additional training, and occasional reports of visual fatigue. Despite these drawbacks, the adoption of 3D laparoscopy appears to support enhanced surgical performance when utilized [25].

The value of 3D imaging is even easier to appreciate when we acknowledge the inroads being made with 3D printing of liver anatomy and tumors and the now decades long quest for 3D intra-operative navigation support via augmented reality (AR) [20, 26–32]. Notably, ICG imaging seems to be superior to current AR solutions, and researchers have begun to hypothesize that it may be necessary to combine ICG with AR to get accurate and useable intra-operative navigation support during liver resection [33, 34].

### The complete robotic surgical system

In larger studies the TRL has potential advantages over other traditional laparoscopic liver resection including slightly higher rates of textbook outcome in liver surgery (TOLS) in an international multi center propensity score matched study [35]. However, a recent metanalysis also found decreased length of stay in patients undergoing laparoscopic liver resection, while fewer transfusions were found in patients undergoing 4 arm robotic assisted liver resection [36]. Nonetheless, the da Vinci Robot (Intuitive Surgical, Sunnyvale, Ca, USA) is still the only FDA-approved truly collaborative robotic system as it has the Integrated Table Motion (ITM) (TRUMPF GmbH + Co. KG, Witzigen, Germany) and the SureForm™ Stapler System (Intuitive Surgical, Sunnyvale, California, USA), which are beyond level 1 Surgical autonomy. As a result, this device currently remains the only robotic platform that is a true cobot.

### The rise of artificial intelligence in surgery

The Internationally Validated European Guidelines on Minimally Invasive Liver Surgery (EGUMILS) organized by Professor Abu Hilal were published in the British Journal of Surgery in 2025 and identified a severe discordance among surgeons regarding the definitions of various minimally invasive techniques for liver resection, specifically, what constitutes robotic-assisted liver surgery [37]. Although surgeons for years have considered the remotely controlled 4 arm CRSS as a surgical robot, earlier versions were just tele-manipulators and, in fact, did not meet the criteria to be considered truly robotic [15].

With the arrival of the age of Artificial Intelligence Surgery (AIS), and therefore, actual examples of intra-operative autonomous actions, surgeons need a better understanding of robotics and updated definitions. Current TRL is really just a form of laparoscopy, with 3D visualization and 4 robotic arms : (1) that holds the camera, (2) enables remotely controlled articulating instruments with 7 degrees of freedom and a final arm that the operating surgeon can also control, albeit the surgeon has to toggle control and cannot control this third arm simultaneously. Notably, there are even problems with the term laparoscopy. Deriving from the Greek, *lapara* meaning flank or side and *skopein* meaning to see, it is not used in many parts of europe. In France and Spain, for example, the term coelioscopic is used instead of laparoscopic with *koilia* coming from the greek word for belly or cavity.

If a 3D laparoscopic camera is held by a robotically-controlled camera holder, and all stapling is done with autonomous handheld GIA staplers and this has the same results as liver resections done with vastly more expensive TRL, perhaps the money saved by not having to buy a CRSS could be used for the creation of AI-powered decision support preoperatively during tumor boards, post-operatively to help enhance the detection of complications and even permit the creation of intra-operative navigation via functional augmented reality (AR) and real-time decision support [38–41]. Nonetheless, the ethics regarding the application of these technologies in surgery and other parts of society still requires much debate and caution as economic and societal implications are still poorly understood [42, 43].

#### 4.1 Limitations

This study has several limitations. First, although propensity score matching (PSM) was used to reduce selection bias, the retrospective design means that unmeasured confounders may still affect the results. The relatively small sample sizes in some groups - particularly the 3D, 1RA, and 4RA groups - limit the ability to detect meaningful differences. The multicenter design of this study, while improving external validity, introduces heterogeneity between centers. The participating institutions were not homogeneous in terms of access to surgical technologies; for example, some centers possessed advanced four-arm robotic systems, while others had only conventional laparoscopy with or without 3D imaging or single-arm cobotic platforms without 3D imaging. Such differences in resources, surgeon experience, and perioperative protocols may have contributed to variability in operative time, blood loss, or hospital stay, beyond the intrinsic effects of each surgical technique. In addition, the analysis focuses on short-term outcomes, leaving long-term oncological results such as disease-free and overall survival unexamined.

Another limitation is the changing definition of robotic surgery, which complicates direct comparisons between minimally invasive approaches. The lack of standardized protocols for robotic-assisted liver resections across centers could also lead to inconsistencies in technique and postoperative care. Finally, the rapid pace of innovation in surgical robotics means that some findings may become outdated as newer systems with greater autonomy and AI integration are introduced. Future research should aim for standardized operative protocols across centers or use multilevel statistical modeling to adjust for institutional clustering. These factors highlight the need for prospective, randomized trials to better determine the advantages of different robotic approaches in liver surgery.

Other limitations include the fact that it is retrospective and spans nearly two decades, during which imaging, anesthesia, and surgical technologies evolved substantially. Although propensity score matching was used to reduce selection bias, residual confounding cannot be excluded. Furthermore, we used pragmatic statistical tests based on data distribution and sample size rather than performing formal normality and variance testing for every endpoint. Advanced models such as Welch ANOVA or mixed-effects regressions could not be implemented with the available resources. As a result, our findings should be regarded as exploratory and hypothesis-generating, warranting confirmation in prospective, multicenter studies with standardized data collection and robust variance-adjusted modeling.

Selection bias is an inherent limitation of this retrospective, multi-center study. The choice of operative approach (Open, DEL, CRL, or TRL) was made by individual surgeons based on patient factors, tumor characteristics, anatomic complexity, and platform availability. These factors may have influenced which patients were considered suitable for MIS approaches and may have favored the selection of less complex cases for DEL, CRL, and TRL. Although we attempted to reduce these effects through standardized data definitions and propensity score matching across key clinical and oncologic variables, residual selection bias cannot be fully excluded. For this reason, all comparisons between operative platforms should be interpreted with caution, especially when subgroup sample sizes are small.

Lastly, this is based on pooled data from multiple centers that differed in access to advanced equipment, robotic platforms, and perioperative protocols. Because the data were historical and lacked uniform center-level identifiers, within-center matching and mixed-effects analyses could not be performed. As a result, equipment and practice pattern differences may bias comparisons between groups. However, consistent inclusion criteria, standardized variable definitions, and data harmonization were applied to minimize this effect. Despite these limitations, the multicenter nature of the study enhances the generalizability of the findings. Future prospective, standardized multicenter collaborations are needed to validate and extend these results.

#### 4.2 Economic implications of platform choice

The economic implications of platform choice are an important consideration in modern minimally invasive liver surgery. Although TRL offers ergonomic and technical advantages, its perceived superiority is often influenced by institutional investment, surgeon preference, and marketing rather than demonstrated clinical benefit. In contrast, DEL and CRL platforms provide similar laparoscopic capabilities at substantially lower cost. Evidence from technology assessments and economic modeling demonstrates that the incremental cost of 3-D laparoscopy is modest when compared with standard 2-D systems, and that platform-related expenses vary substantially across imaging technologies [44]. In addition, recent cost analyses of robotic liver surgery confirm that TRL carries significantly higher procedural costs than conventional MIS approaches because of instrument expenses, amortization of the robotic platform, and maintenance contracts [45]. These considerations highlight the importance of aligning platform selection with both clinical outcomes and institutional resource constraints.

Although a formal cost analysis was not included in this study, the published literature consistently demonstrates meaningful differences in procedural costs across these platforms. Standard and 3-D laparoscopy generally add 150 to 600 USD per case, depending on equipment and disposable requirements [44]. Cobot-assisted approaches that use a single robotic arm for scope stabilization typically add 300 to 800 USD per case. In contrast, TRL is associated with significantly higher per-case costs, commonly reported as 3,000 to 8,000 USD above standard laparoscopy [45]. Including these descriptive ranges provides a clearer picture of the real-world economic impact of each modality and supports the interpretation of our findings within a resource-conscious framework.

## 5. Conclusions

This multi-center study compared five surgical approaches for liver resection in colorectal metastases, demonstrating that minimally invasive techniques—particularly laparoscopy with robotic assistance—offer advantages in blood loss and hospital stay over open surgery. However, the benefits of TRL over simpler robotic-assisted methods (CRL) or DEL remain unclear, with longer operative times and no significant improvements in key outcomes. Nevertheless, analyses involving DEL, CRL and TRL groups are underpowered and considered exploratory. These findings are hypothesis-generating and larger prospective studies are needed to see if cost-effective, modular robotic enhancements (e.g., autonomous camera holders or staplers) with and without 3-D digital enhancement may provide comparable benefits to complex robotic platforms, and thus, allow resources to be redirected toward AI-driven surgical innovations. As a result, future clinical studies should focus on prospective, randomized trials with standardized protocols to further evaluate these evolving technologies.

It must be acknowledged that current telemanipulation surgery is the ideal way for industry to one day create truly robotic surgery, level 5, where no human is involved in the procedure. Current “robotic” surgeons could be sowing the seeds of their own destruction. Because of this, surgeons must continue to assess more collaborative forms of robotic surgery, specifically CRL +/- DEL. It could be that cobotics is the best way to keep the surgeon in the loop. It must be recalled that the first Grandmaster to lose to a chess competition to a computer, Gary Kasparov, noted that when a machine competed against a human in chess that the surgeon wins, and that when a computer competes against another computer there is no clear winner. However, when a man and computer join forces, they are superior to a computer alone. It is because of this observation and the findings in this study that we call for increased research in collaborative robotics with digital augmentation in surgery.

## Data Availability

Data available upon reasonable request.
